# Comparison of Emotional Content in Text Responses From Physicians and AI Chatbots to Patient Health Queries: Cross-Sectional Study

**DOI:** 10.2196/85516

**Published:** 2026-03-06

**Authors:** Daniel T Burns, Channing Bice, Paul E Johnson, Nicholas Chia, Timothy Robinson

**Affiliations:** 1 Department of Mathematics & Statistics University of Wyoming Laramie, WY United States; 2 Department of Journalism & Media Communication Colorado State University Fort Collins, CO United States; 3 Department of Otolaryngology- Head and Neck Surgery University of Washington Seattle, WA United States; 4 Data Science and Learning Argonne National Laboratory Lemont, IL United States

**Keywords:** responses, emotional, chatbot, physicians, health, ChatGPT, artificial intelligence, AI

## Abstract

**Background:**

Surveys show that many people are willing to use generative artificial intelligence (AI) for health questions. Prior research has largely focused on chatbot accuracy, with some studies finding that both physicians and consumers overwhelmingly prefer chatbot-generated text over physician responses.

**Objective:**

This study aimed to characterize and compare the emotional content of responses from physicians and 2 AI chatbots (OpenAI’s ChatGPT and Google’s Gemini) and to assess differences in reading level and use of medical disclaimers.

**Methods:**

A public, patient-deidentified telehealth website was used to compile 100 physician-answered questions. The same questions were posed to both chatbots between May 18 and 19, 2025. Two coders classified the emotional content of each sentence using a predefined codebook and reviewed for agreement. Emotions were ranked as primary, secondary, and tertiary by the proportion of sentences classified as each emotion per response. Multinomial logistic regression compared emotional rankings using physician responses as the reference. Word count, Flesch Reading Ease, and Flesch-Kincaid Grade Level were analyzed via ANOVA with the Tukey honestly significant difference test. Disclaimer use was compared between chatbots using a *χ*^2^ test.

**Results:**

Primary emotions were overwhelmingly neutral, except for one response from each chatbot in which anger was primary. For secondary emotions, the odds ratio of hope was 80.28% (95% CI 37.71%-93.76%) lower for ChatGPT, while the odds ratio of fear was 3.29 (95% CI 1.44-7.49) times higher for Gemini. For tertiary emotions, the odds ratio of compassion was 1.94 (95% CI 1.06-3.54) times higher, and the odds ratio of having no tertiary emotion was 84.33% (95% CI 64.72%-93.04%) lower for Gemini. Gemini responses averaged 889.1 (SD 305.7) words, ChatGPT 476.5 (SD 109.5), and physicians 193.5 (SD 113.6). Gemini had the lowest average Flesch Reading Ease score at 39.9 (SD 8.8), followed by ChatGPT at 45.8 (SD 12.8), while physicians had the highest at 51.9 (SD 13.6). Gemini had the highest average Flesch-Kincaid Grade Level at 11.3 (SD 1.5), followed by ChatGPT at 9.9 (SD 1.9), and physicians at 9.2 (SD 2.4). Gemini was significantly more likely to include a disclaimer than ChatGPT (*χ*^2^_1_=49.2; *P*<.001).

**Conclusions:**

Chatbot responses were significantly (*P*<.001) longer and more difficult to read than physician responses and were more likely to contain a wider range of emotions. Qualitatively, chatbot responses were more varied in their presentation as well as in the breadth of the emotions themselves. The findings of this study could be used to inform more emotionally connected physician responses to patient message queries.

## Introduction

Health communication extends beyond facts to include emotional reassurance, persuasion, and support, all of which are central to how people make health decisions. As more people turn to the internet for health information, generative artificial intelligence (GenAI) chatbots have rapidly emerged as influential sources for medical guidance, self-diagnosis, and emotional support. Research shows that many users, including physicians, often rate chatbot responses as accurate, empathetic, and useful, though concerns remain about misinformation, ethics, and accountability. Internet-driven health information seeking continues to grow. A report published in 2023 by the US Centers for Disease Control and Prevention found that 58.5% of adults surveyed in the second half of 2022 said they used the internet to look for health or medical information [1]. Between the time of data collection and publication of that study, GenAI chatbots began to be released for widespread public use, starting with ChatGPT (version 3.5; OpenAI) in November 2022. Subsequently, a 2023 study collected responses from 607 participants, of which 476 (78.4%) said that they were willing to use ChatGPT for self-diagnosis [2], while a 2025 study found that 21.2% (63/297) of respondents actively used chatbots for health information seeking [3].

Multiple studies have evaluated the accuracy of responses from chatbots to health-related questions across topics such as cancer, emergency medicine, general inquiries, and symptom checking. The results of these studies ranged from generally quite good (for symptom checking and general inquiries) to fair regarding the accuracy of information provided and were mixed in terms of dangerous or misinformative responses (no such responses in a cancer study and up to 35% of responses in an emergency medicine study) [4-7]. Beyond response accuracy, research has found that physicians prefer responses crafted by chatbots more than responses from other physicians 78% of the time [6]. Additionally, both patients and physicians have ranked ChatGPT responses significantly higher in empathy and usefulness than physician responses [8]. Recent studies have revealed that although health care professionals have favorable opinions regarding chatbot efficiency, they raise concerns about ethics and accountability, while the public appreciates the accessibility and emotional support provided by chatbots [9].

Health communication is a multidisciplinary field that integrates concepts from media studies, psychology, sociology, anthropology, informatics, education, and medical sciences [10,11]. It encompasses a variety of spoken, written, and computer-mediated texts and processes [12,13]. Health communication plays a crucial role in delivering health information to a variety of audiences, thereby enhancing patient and health care professional or medical institution understanding of health risks and guiding them in prevention, detection, diagnosis, and treatment [14]. Health communication can come from diverse sources, including health care professionals, family members, peer support groups, health education materials, news articles, and online resources [15,16].

Beyond factual information, individuals also seek emotion-based information to address the emotional dimensions of health concerns [17]. Feelings such as fear, anxiety, worry, and shame often accompany health-related decision-making, making emotional support, empathy, and encouragement critical components of health communication [18]. Supportive and emotionally responsive information has been shown to alleviate health-related stress, worry, and depression, while also enhancing individuals’ commitment to managing health risks [14,19,20].

Studies have examined users’ perceptions of chatbots, including their likability, capacity to build rapport, and ability to demonstrate empathy [13,21,22]. Recent studies have also demonstrated the persuasiveness of chatbots, including their ability to reduce belief in conspiracy theories and to change people’s minds more broadly [23-25]. The increase in willingness to use the internet and GenAI for health information seeking corresponds with a rise in electronic patient portal messaging, particularly in the wake of the COVID-19 pandemic, and the increased burden this places on physicians and health care services [26-29]. Thus, the aim of this study was to investigate key differences in the emotional support and readability of health information offered by physicians and the commonly used artificial intelligence (AI) chatbots ChatGPT and Gemini (version 2.0 Flash; Google). We describe the emotional content and answer complexity contained within text responses to a variety of health questions across medical specialty topics, as posted on an online telehealth website.

## Methods

### Data Acquisition and Preparation

Following similar data collection methods as previously described [30-34], data were obtained from iCliniq (Orane Healthcare India Private Limited), an international telehealth website with publicly accessible question-and-answer forums where users can pay to ask verified physicians questions. These anonymized questions are categorized by condition and medical specialty by the website. For this study, questions and answers were selected from a subset of 10 medical specialties. From these 10 specialties, 10 question-answer pairs were randomly selected per specialty, resulting in a total of 100 pairs. The 10 specialties were allergy specialist, dermatology, family physician, general practitioner, internal medicine, medical oncology, neurology, pediatrics, psychiatry, and urology. Questions from this website were chosen using stratified random sampling (with medical specialties as strata) and subsequently posed to chatbots, and responses were compared with those of the responding physicians. In cases where the question elicited a dialogue (ie, back-and-forth discussion beyond the first question and response), only the initial question and answer were used. We next removed greetings and stock response openings from the questions and answers to reflect a more natural conversational tone, especially for the questions, to ensure that the chatbot responses were not unduly influenced. For example, phrases such as “Hello doctor,” “Hi doctor,” “Dear doctor,” and “Doctor,” were removed from the patient questions, while phrases such as “Hi, Welcome” and “Hello, Welcome back” were removed from the physician responses. Responses were copied to a text editor (eg, a Microsoft Word or PDF document) for downstream classification analysis.

### AI Chatbots

Two chatbots were used for comparison with physician responses: ChatGPT (GPT-4o) and Gemini 2.0 Flash. For each chatbot, each question (prompt) was asked in a fresh session, with “Memory” (chat history) option set to off so that no previous question or response impacted a subsequent interaction. Gemini was queried via its application programming interface from a custom Python script on May 18, 2025. As ChatGPT offered no free application programming interface access, questions were manually copied and pasted into the ChatGPT user interface on its website, and responses were similarly copied and pasted into a text editor. ChatGPT was queried from May 18 to May 19, 2025, as rate limits inhibited the ability to ask all 100 questions on the same day. The chatbots were given no special instructions, and no prompt engineering was applied to mimic real-world patient use [35-39]. Questions were asked under the default settings of each chatbot, and chatbots were free to respond however they saw fit based on their settings.

### Response Summaries

Word count was calculated for all responses using the R package *stringi* [40]. Flesch Reading Ease (FRE) and Flesch-Kincaid Grade Level (FKGL) are commonly used scores for assessing the readability of written materials [41-45]. The FRE score uses a formula containing total words, total sentences, and total syllables to calculate a score that indicates the reading difficulty of the material, with higher scores indicating easier material (eg, scores of 90-100 are considered very easy to read and understandable by an average 11-year-old, while scores of 30-50 are considered difficult to read and understandable by someone in college). The FKGL formula uses the same parameters as the FRE formula but calculates a score as a US grade level (or number of years of education needed) for judging readability. FRE and FKGL scores were calculated using Microsoft Word’s built-in summaries. According to the National Library of Medicine, nearly 9 out of 10 adults struggle with health literacy [46]. The US Department of Education reported that 54% of Americans aged 16 to 74 years read below a sixth-grade level [47]. The American Medical Association recommends that patient education materials be written at or below a sixth-grade level [48], while the National Institutes of Health recommends it to be at or below an eighth-grade level [41,49].

To get a better understanding of how these chatbots communicate about health-related topics, their use of “not a doctor” disclaimers was also assessed. In this context, a disclaimer consisted of any version of the chatbot explicitly stating that it is not a physician or medical professional, that the information it provided did not constitute medical advice, or that it was an AI and could not provide medical advice. This variable was recorded as 1 for “yes” and 0 for “no” depending on whether a response contained such a disclaimer.

### Emotion Classification

A quantitative content analysis was conducted to systematically classify the emotional content embedded within physician and chatbot responses. Defined as “a research technique for the objective, systematic, and quantitative description of the manifest content of communication” [50], this method allowed us to categorize and quantify textual features to identify patterns and frequencies in the data. The codebook was developed using a robust body of literature in psychology and media communication to conceptualize and define each variable of interest (Table 1). The codebook contained 6 emotions (anger, compassion, fear, guilt, hope, and sadness) plus neutral or rational. For brevity, we summarize the codes, definitions, and associated literature in Table 1. The first and second authors (DTB and CB) served as the coders for this study. The unit of analysis (ie, the content coded as a part of our study) comprised the sentences and phrases of each response. The coders used MAXQDA (version 24.10; VERBI GmbH) to conduct the coding and generate classification counts. These counts were then used to rank the top 3 emotions expressed in each response (ie, primary, secondary, and tertiary), using MAXQDA’s Document Portrait function (refer to Multimedia Appendix 1 or “Document Portrait” entry of the MAXQDA Manual [51] for details).

**Table 1:** Emotion classification codebook.

Emotion	Definition	Examples	References
Anger	The response contains an emotion characterized by tension or hostility arising from frustration or the belief that an injury or injustice has been committed against oneself or an important other. It can manifest itself in behaviors designed to remove the object of anger.	“My doctor told me that my symptoms were ‘all in my head’ and now I have cancer that has progressed to stage 4, when it could have been caught earlier.” “It’s so frustrating that your concerns aren’t being taken seriously, that’s unacceptable!”	[52-54]
Compassion	The response contains an emotion characterized by feelings of empathy in response to witnessing the pain or discomfort of others. It can manifest as a desire to help alleviate pain or discomfort and to engage in helping or sharing behaviors.	“I can help you find a nearby dermatology clinic.” “I’m really sorry to hear that you’re feeling concerned about this. It’s understandable to be worried, but based on what you’ve described, there are a few possible explanations that might be less serious than cancer. That said, it’s still important to get it checked out.”	[55,56]
Fear	The response contains an emotion characterized by feelings of worry that is typically triggered by detecting an imminent threat. It can manifest itself as behaviors designed to move away from or avoid the source of fear.	“COVID-19 can be lethal.” “Applying bleach to a skin lesion can be dangerous.” “Swallowing a toothpick can be dangerous and may lead to serious health risks, though the actual risk of dying depends on a variety of factors, such as the size and shape of the toothpick, how it travels through the digestive system, and whether it causes complications.”	[52-54]
Guilt	The response contains a self-conscious emotion characterized by an appraisal of having done (or thought) something that is wrong or violates internalized or socially acceptable standards of behavior. It can manifest itself as behaviors designed to undo or mitigate the wrong.	“If you have COVID-19, don’t go to work or school—stay home to protect others.” “I should have taken my child to the doctor sooner.”	[52-54]
Hope	The response contains a prospect-based emotion experienced as a desire for a better future, despite current uncertainty or unfavorable circumstances. It manifests itself as behaviors designed to move toward their goals by taking action and/or planning for the future.	“Although your lesion looks bad now, consistently applying Vaseline will help prevent infection and scarring.” “We caught the condition early, so many good treatment options are available.”	[53,57]
Sadness	The response contains an emotion characterized by sorrow, loss, resignation, or the absence of a desired reward. It manifests itself through a lack of motivation or actions to change one’s circumstances.	“I’m tired of trying new depression medications. Nothing is helping.” “I can barely get out of bed I feel so hopeless.”	[53,58]
Neutral or rational	The response does not contain an emotion but offers fact-based and verifiable information, definitions, or advice about the health condition that appeals to one’s reason or rational thought processes.	“Since the cough has been persisting for weeks, and it’s affecting your ability to sleep, it would be a good idea to consult a health care professional to rule out more serious issues like pneumonia, bronchitis, or any other lung concerns.” “Sounds like you will be fine. You should flush the eye anytime you get a chemical or foreign body in the eye. You can also contact Poison Control 1-800-222-1222.”	[54,59]

To ensure consistency, the authors discussed the conceptual and operational definitions of each emotion and reviewed sample responses. We then independently coded a practice set of 10 responses to verify the codebook’s applicability. Through regular meetings and discussions, the 2 coders compared the categorization of responses, resolved discrepancies, and revised and modified the codebook. An example of a coded item and document portrait is presented in Multimedia Appendix 1. Intercoder reliability was assessed using a stratified (by medical specialty) random selection of 50 responses independently evaluated by the 2 coders, followed by 2 rounds of joint review. Reliability was calculated using Cohen κ coefficient, which takes chance agreement into account [60]. Initially, Cohen κ ranged between 0.64 and 0.81. Coefficients ≥0.8 were considered reliable [61], and following the second round of review, Cohen *κ* ranged between 0.81 and 0.86.

### Statistical Analysis

All statistical analyses were performed using R (version 4.5.1; R Foundation for Statistical Computing) [62]. For the readability-related response summaries, ANOVAs followed by Tukey’s honest significant difference test were used for pairwise comparisons. “Not a doctor” disclaimer use between the 2 chatbots was compared by a χ^2^ test. Multinomial logistic regression was used to compare emotion classification results, with physicians set as the reference group.

For the secondary emotion analysis, 39 responses were removed (13 from each responder) because, for each of these 13 questions, the physician’s responses contained only one (primary) emotion, necessitating “none” as the classification for the secondary and tertiary emotions. With the exception of one Gemini response, the chatbots did not have any “none” secondary classifications, and thus, coefficient estimation was highly biased and unreliable. Similarly, 9 responses (3 from each responder) were removed from the tertiary emotion analysis, as Gemini was the only responder to have anger classified as a tertiary emotion. Statistical significance was set at *P*<.05 for all tests.

### Ethical Considerations

This study analyzed publicly available data from a freely accessible telehealth website. No personal or identifiable patient or physician data were collected or used. According to the US Department of Health and Human Services, 45 Code of Federal Regulations 46.102(e)(1) [63], this study does not meet the definition of human participants research and therefore is exempt from institutional review board review.

## Results

### Response Summaries

**Figure 1:**
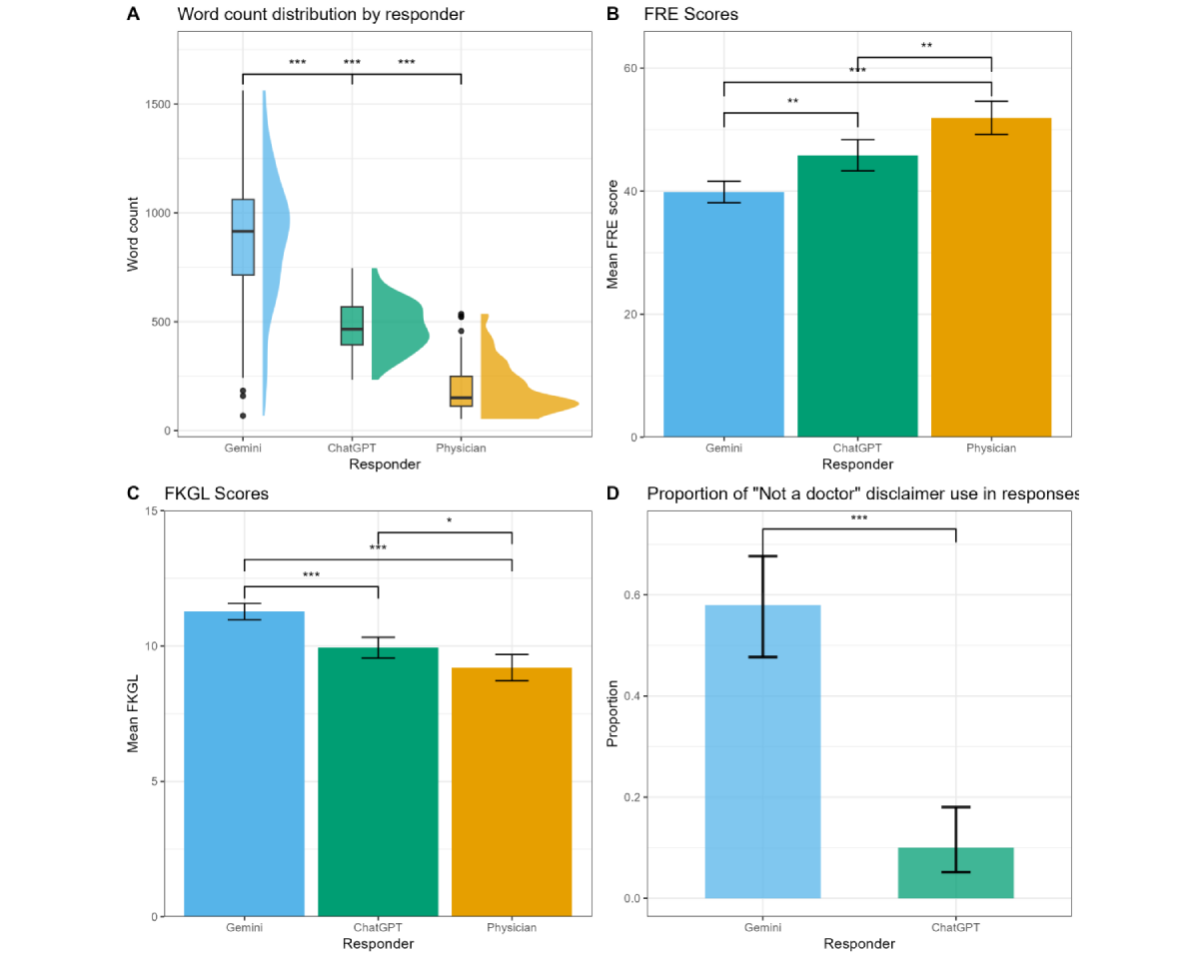
Comparison of response length, readability, and disclaimer use among physicians, ChatGPT, and Gemini. (A) Boxplots, with accompanying density plots, of the word count distributions for each responder. The bold black line inside boxes represents the median, and black circles denote outliers. (B) Mean Flesch Reading Ease (FRE) scores per responder. Higher scores indicate greater readability. (C) Mean Flesch-Kincaid Grade Level (FKGL) scores per responder. Higher scores indicate a higher educational level required for reading comprehension. (D) Proportion of responses that contained disclaimer use for each chatbot. Differences among responders in A-C were calculated via ANOVA followed by the Tukey honestly significant difference test. Differences among responders in D were calculated via the chi-square test. Error bars in B-D represent 95% CIs. *P=.03, **P=.001, ***P<.001.

Figure 1A shows the distribution of word counts for each responder. All 3 responders’ word counts were significantly different from one another (*F*_2,297_=310.1; *P*<.001). Gemini had the highest word count, with an average of 889.1 (SD 305.7) words per response, followed by ChatGPT with an average of 476.5 (SD 109.5) words per response, and physicians with the lowest average at 193.5 (SD 113.6) words per response.

All 3 responders’ FRE scores were significantly different from one another (*F*_2,297_=27.5; *P*<.001). As shown in Figure 1B, Gemini had the lowest average FRE score at 39.9 (SD 8.8), followed by ChatGPT at 45.8 (SD 12.8), while physicians had the highest average score at 51.9 (SD 13.6). All 3 responders’ FKGL scores were also significantly different from one another (*F*_2,297_=25.7; *P*<.001). Figure 1C shows that Gemini had the highest average FGKL score of 11.3 (SD 1.5), followed by ChatGPT with an average score of 9.9 (SD 1.9), and physicians again having the easiest readability of the 3 groups, with an average FGKL score of 9.2 (SD 2.4).

As shown in Figure 1D, disclaimer use differed significantly between the 2 chatbots (*χ*^2^_1_=49.22; *P*<.001). Gemini made such a statement 58% (58/100; 95% CI 47.71%-67.67%) of the time compared with ChatGPT’s 10% (10/100; 95% CI 5.16%-18.04%).

### Emotion Classification

Neutral or rational was the most common emotion expressed across all 3 responders. It was the primary emotion for all 100 (100%) of the physician responses and for 99 (99%) out of 100 for each chatbot. In the one response per chatbot in which neutral was not the primary emotion, fear was the most prominent emotion. In both instances, the chatbots directed the question asker to seek immediate medical attention, as they deemed the situations to be very concerning.

All comparisons for secondary and tertiary emotions were made relative to physician responses (ie, the physician group was set as the reference and the chatbots were compared with it). For secondary emotions (Tables 2 and 3), the odds ratio of a hope response was 80.28% (95% CI 37.71%-93.76%; *P*=.006) lower for ChatGPT. The odds ratio of a fear response was 3.29 (95% CI 1.44-7.49; *P*=.005) times higher for Gemini. Physician responses did not have a secondary emotion 13% (13/00) of the time (ie, the entire response was classified as containing only one emotion), while Gemini had such responses 1% (1/100) of the time, and ChatGPT always had at least 2 emotions classified in its responses. Physicians never had anger classified as a secondary emotion, while Gemini and ChatGPT each did so once.

For tertiary emotions (Tables 4 and 5), the odds ratio of a compassion response was 1.94 (95% CI 1.06-3.54; *P*=.03) times higher, and the odds ratio of having no tertiary emotion classified (none) was 84.33% (95% CI 64.72%-93.04%; *P*<.001) lower for Gemini. The odds ratio of having no tertiary emotion classified (none) was 53% (95% CI 3.42%-78.51%; *P*=.06) lower for ChatGPT. Gemini was the only responder to have anger classified as a tertiary emotion.

**Table 2:** Secondary emotions by responder (N=100)^a^.

Emotion	Physician, n (%)	ChatGPT-4o, n (%)	Gemini Flash 2.0, n (%)
Anger	0 (0)	1 (1)	1 (1)
Compassion	57 (57)	80 (80)	51 (51)
Fear	10 (10)	11 (11)	33 (33)
Guilt	3 (3)	2 (2)	3 (3)
Hope	17 (17)	5 (5)	10 (10)
Neutral	0 (0)	1 (1)	1 (1)
None	13 (13)	0 (0)	1 (1)
Sadness	0 (0)	0 (0)	0 (0)

^a^Responses from each responder containing the labeled emotions at the secondary rank. Each responder was asked the same 100 questions.

**Table 3:** Multinomial logistic regression results for secondary emotions^a^.

Responder and emotions		Odds ratio (95% CI)	SE	Wald Z	*P* value
**ChatGPT**					
	Compassion	1.268 (0.893-1.800)	0.179	1.329	.18
	Fear	0.710 (0.270-1.866)	0.493	−0.695	.49
	Guilt	0.263 (0.027-2.597)	1.168	−1.143	.25
	Hope	0.197 (0.062-0.623)	0.587	−2.766	.006
**Gemini**					
	Compassion	0.821 (0.556-1.213)	0.199	−0.989	.32
	Fear	3.287 (1.442-7.491)	0.420	2.831	.005
	Guilt	1.217 (0.234-6.321)	0.840	0.234	.81
	Hope	0.685 (0.277-1.693)	0.462	−0.820	.41

^a^Transformed (exponentiated) results of multinomial logistic regression comparing the secondary emotion rankings of the 2 chatbots to physicians.

**Table 4:** Tertiary emotions by responder (N=100)^a^.

Emotion	Physician, n (%)	ChatGPT (GPT-4o), n (%)	Gemini Flash 2.0, n (%)
Anger	0 (0)	0 (0)	3 (3)
Compassion	17 (17)	20 (20)	31 (31)
Fear	10 (10)	18 (18)	12 (12)
Guilt	2 (2)	6 (6)	5 (5)
Hope	13 (13)	22 (22)	32 (32)
Neutral	0 (0)	0 (0)	0 (0)
None	58 (58)	34 (34)	17 (17)
Sadness	0 (0)	0 (0)	0 (0)

^a^Responses from each responder containing the labeled emotions at the tertiary rank. Each responder was asked the same 100 questions.

**Table 5:** Multinomial logistic regression results for tertiary emotions^a^.

Responder and emotion		Odds ratio (95% CI)	SE	Wald Z	*P* value
**ChatGPT**					
	Compassion	1.250 (0.648-2.412)	0.335	0.665	.51
	Fear	1.280 (0.458-3.577)	0.524	0.471	.64
	Guilt	2.400 (0.425-13.589)	0.883	0.992	.32
	Hope	1.354 (0.524-3.500)	0.485	0.625	.53
	None	0.471 (0.215-1.034)	0.401	−1.876	.06
**Gemini**					
	Compassion	1.937 (1.060-3.542)	0.308	2.149	.03
	Fear	0.619 (0.220-1.741)	0.527	−0.908	.36
	Guilt	1.290 (0.225-7.405)	0.891	0.286	.78
	Hope	1.270 (0.525-3.072)	0.450	0.531	.60
	None	0.157 (0.070-0.353)	0.414	−4.477	<.001

^a^Transformed (exponentiated) results of multinomial logistic regression comparing the tertiary emotion rankings of the 2 chatbots to physicians.

## Discussion

### Principal Findings

Overall, the AI chatbots provided longer and more detailed responses than physicians. Whether the additional detail provided in chatbot responses was appropriate or correct was not investigated in this study. While initial physician responses were often a series of questions, presumably akin to a history-taking for gathering more information for a proper diagnosis or suggestion, chatbots often presented lists of possible explanations that were ranked from most to least likely. The chatbots also often asked follow-up or clarification questions, but rarely at the expense of providing answers; chatbot responses were combinations of detailed lists and explanations, with occasional clarifying questions interspersed. This additional feature provided by the chatbots could also help explain why the responses were calculated to be more difficult to read. Although all responders provided answers well above the national average reading grade level [47], the chatbots were more difficult to read and understand, which may negate some of the usefulness of the added information, as it could be overly complex for a typical patient to understand and therefore properly act on.

Similarly, the very infrequent use of disclaimers, particularly by ChatGPT, explicitly stating that chatbots are not medical professionals or that the information provided does not replace consultation with a qualified clinician may place users at risk. If the information is presented in a way that is difficult to understand, users could find it challenging to discern useful information from irrelevant or potentially harmful information. This problem is further compounded by chatbots’ tendency to provide responses in a manner that conveys strong certainty and expertise in the content [64,65].

The bulk of the responses from all 3 responders were neutral in emotional content, as the vast majority consisted of factual explanations and definitions. Physicians were more likely to provide a response that contained only one emotion (neutral), perhaps as a consequence of their generally much shorter responses, which often consisted of follow-up questions. In contrast, the chatbots had a relatively formulaic response pattern, wherein they opened with an emotional statement (usually compassion or fear) acknowledging the situation of the question asker. Similarly, the chatbots ended their responses with summaries of the overall response and further emotional appeals (usually compassion, fear, or hope).

Although compassionate content proportion did not differ significantly between the chatbots and physicians in a quantitative sense, the quality of the emotions presented was noticeably different. For example, a compassionate statement from physicians often looked like “I understand your concern,” “I hope this helps,” or “I read your query and can understand your concern,” while compassionate statements from the chatbots included “Let’s break down the problems and discuss potential solutions,” “It’s completely understandable that you’re feeling lost and frustrated,” “I’m really sorry you’re going through [this situation]. Here’s a structured overview to help guide you through managing your condition and exploring safer, longer-term strategies,” and “You’re absolutely right to be concerned, and it’s great that you’re actively looking into this. Let’s go over the key points in your situation and steps you can take.” Previous research has shown that patients, physicians, and third-party evaluators consistently perceive AI to be more empathetic and compassionate than physicians [8,66-68]. Our results lend support to these findings and help to show that this difference comes from the quality and content of the written responses rather than from the frequency or proportion of an emotion being conveyed. Similar differences in quality of emotion in the responses were present for other emotions as well. These increases in empathy, sympathy, and emotional support may also play a role in why people consistently prefer responses from chatbots over those from physicians [69].

However, further research is needed to explore how the emotional tone of chatbot responses influences individuals’ health information–seeking behaviors and health information–processing behaviors, which in turn affect their engagement with health-related actions. These results, coupled with the previously mentioned patient and physician preferences for chatbot responses, warrant an investigation into possible reasons for these effects. As good communication skills are increasingly viewed as an important aspect of health care training and practice [70-73], these results can be used to inform physicians’ use of chatbots as a tool to better help connect with patients in a more empathetic manner. Future research should investigate optimizing prompting strategies for chatbot-assisted message drafting to determine patient perceptions, as well as quality and safety outcomes.

### Limitations

There are several limitations to this study. Importantly, a key limitation is the inability to exactly replicate the results. This limitation is due to the ever-changing nature of the AI models used for these chatbots; the models get updated frequently, often without any detail regarding when or what changes were made. Often, these updates fundamentally change the way that chatbots engage with and respond to users. The behavior of the 2 chatbots used in this study, chosen for their popularity and free access, may not be representative of all chatbot services that are currently available.

Another limitation is potential cultural norm disparities. The authors are all from the United States, where both chatbots were accessed and created or hosted by US companies. This factor could cause unintended bias in emotion classification, as the expectations and definitions in one culture may not be the same as those in another. Furthermore, because the website is publicly accessible, physicians responding to questions may have access to patient information that is not visible on the question page and therefore not captured in the dataset. This access feature could complicate the interpretations of this study, as it is possible that physicians gave short, emotionally neutral answers because they had more information than was provided in just the question alone, which the researchers and therefore the chatbots would be unaware of.

Another potential limitation is that the patient questions used were chosen randomly; however, the authors selected the medical specialty topic areas containing the questions. Although the intention was to ensure an array of question types across various topics, it is possible that this approach inadvertently biased results if the selected questions were too similar. Additionally, for the sake of simplicity and feasibility, our focus was limited to 7 emotions, classified by 2 of the authors. Although these were selected from health communication literature and deemed useful and informative for the purposes of this study, it is possible that different emotions, or combinations of emotions, could yield different conclusions as well. For similar reasons, we also limited the selected AI chatbots to 2 free options, which left out popular alternatives, such as Anthropic’s Claude, Microsoft’s Copilot, and High-Flyer’s DeepSeek, thus limiting the scope of this study and its conclusions.

### Conclusions

This study demonstrates key differences in the emotional support and readability of health information offered by physicians and AI chatbots (ChatGPT and Gemini). It also extends the literature examining the usefulness of AI-generated drafts by demonstrating chatbots’ ability to craft emotionally connective responses, potentially enhancing emotional support for patients, while mitigating physician burnout and compassion fatigue [74]. Compared to physicians, the AI chatbots’ responses were much longer and more difficult to read. ChatGPT rarely included a disclaimer in its responses indicating that it is not a medical professional (only 10% of responses), while Gemini included one more often (60% of responses).

Although few statistically significant differences existed between the emotional content of physicians and chatbots, there were substantial qualitative differences in the depth and breadth of emotionality presented, especially for compassion (empathy). This finding could be due, in part, to the preferential training of chatbots to be more personable and agreeable, coupled with the fact that they do not suffer from burnout or fatigue.

An often overlooked aspect of AI-generated responses is the variability or uncertainty that is inherent in the models. All generative AI responses are probabilistic responses, which implies uncertainty can be investigated. However, approaches for assessing that uncertainty remain an unanswered research question; although beyond the scope of this study, this represents an important direction for future research.

## Appendix

Multimedia Appendix 1 Text emotion classification and ranking. (A) Example physician response with sentences highlighted for the coded emotion (gray=neutral or rational, purple=compassion, and orange=fear). (B) Document Portrait classification summary of the coded response in (A), showing the proportion of each emotion in descending order from left to right. In this example response, neutral was coded the most, followed by compassion, then fear. Classifications and document portrait summaries were carried out in MAXQDA software.

## Abbreviations

AI: artificial intelligence
FKGL: Flesch-Kincaid Grade Level
FRE: Flesch Reading Ease
GenAI: generative artificial intelligence

## Data Availability

The datasets generated or analyzed during this study are available from the corresponding author on reasonable request.
