# Supplementary figures and images for: Comparison of Emotional Content in Text Responses From Physicians and AI Chatbots to Patient Health Queries: Cross-Sectional Study
Source: J Med Internet Res. 2026 Mar 6;28:e85516. doi: 10.2196/85516 (PMC13005063; doi:10.2196/85516)

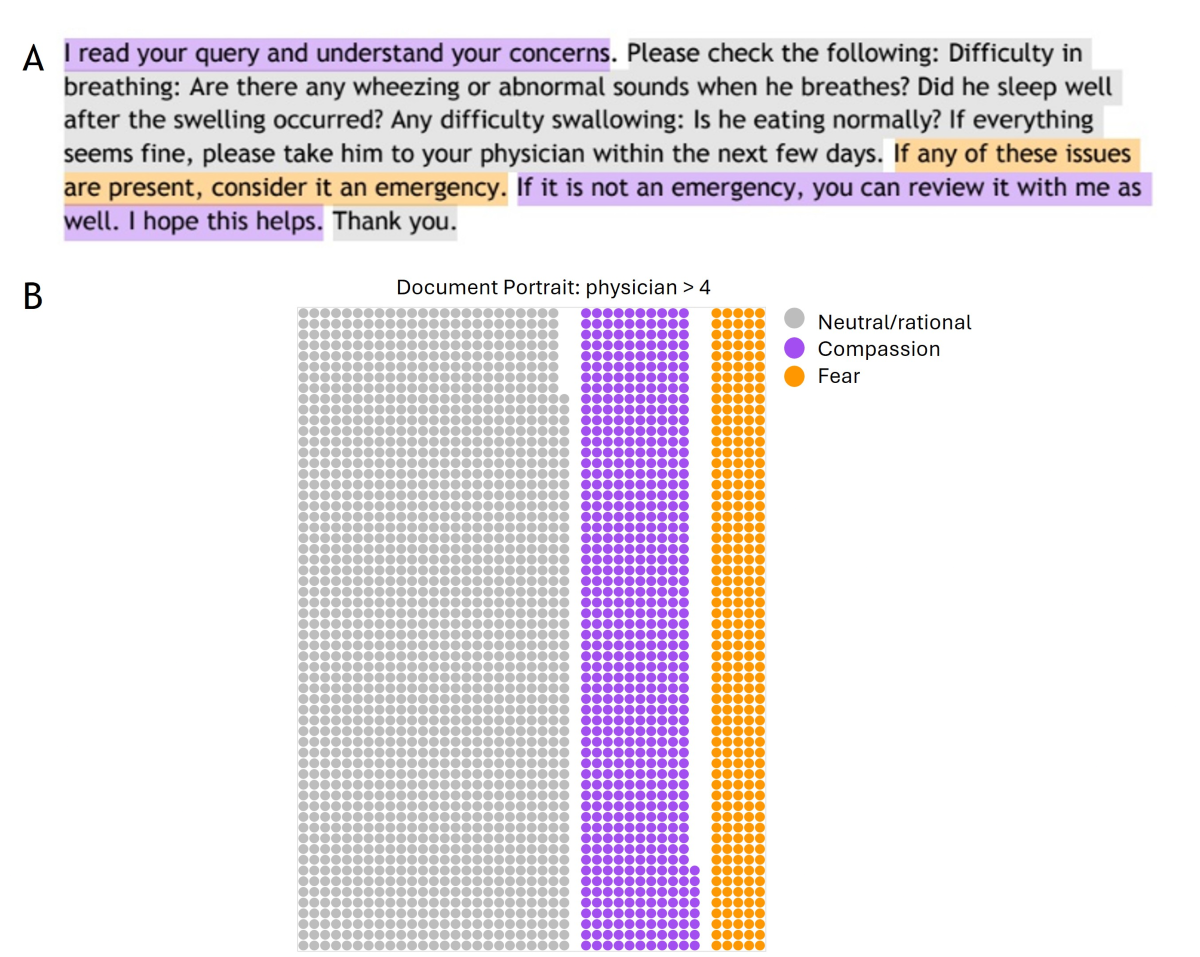

Supplement: Multimedia Appendix 1 [file jmir_v28i1e85516_app1.png]
